# Synthesis of Piperidine Nucleosides as Conformationally Restricted Immucillin Mimics

**DOI:** 10.3390/molecules26061652

**Published:** 2021-03-16

**Authors:** Maria De Fenza, Anna Esposito, Daniele D’Alonzo, Annalisa Guaragna

**Affiliations:** 1 Department of Chemical Sciences, University of Naples Federico II, Via Cintia, 80126 Naples, Italy; maria.defenza@unina.it (M.D.F.); anna.esposito5@unina.it (A.E.); dandalonzo@unina.it (D.D.); 2 Department of Chemical, Materials and Production Engineering, University of Naples Federico II, Piazzale V. Tecchio 80, 80125 Naples, Italy

**Keywords:** immucillins, nucleoside analogues, de novo synthesis, iminosugars, biomimetics, conformationally restricted nucleosides, piperidine nucleosides, polymer-supported triphenyl phosphine

## Abstract

The de novo synthesis of piperidine nucleosides from our homologating agent 5,6-dihydro-1,4-dithiin is herein reported. The structure and conformation of nucleosides were conceived to faithfully resemble the well-known nucleoside drugs Immucillins H and A in their bioactive conformation. NMR analysis of the synthesized compounds confirmed that they adopt an iminosugar conformation bearing the nucleobases and the hydroxyl groups in the appropriate orientation.

## 1. Introduction

**Figure 1:**
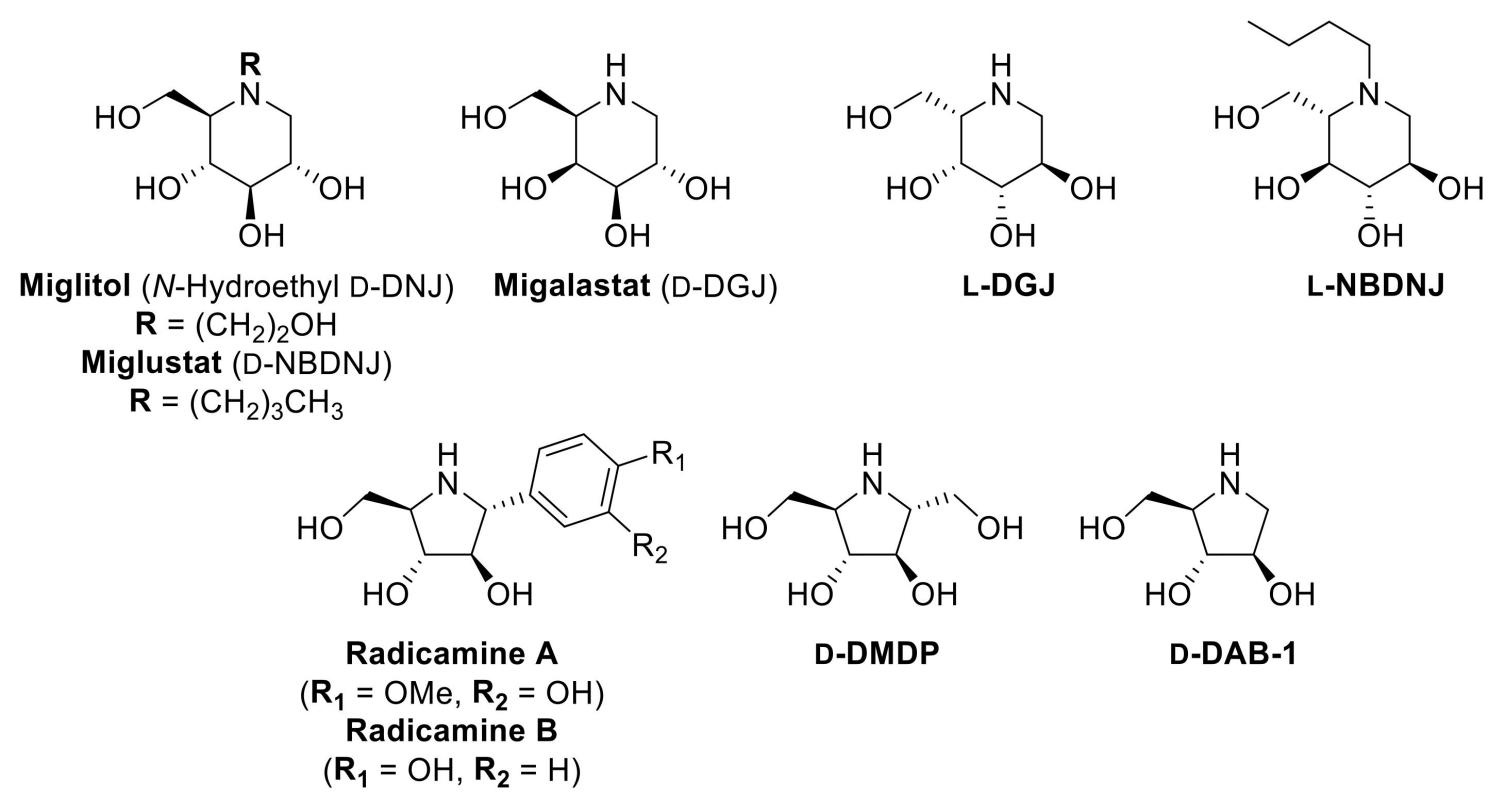
Pyrrolidine and piperidine iminosugars.

**Figure 2:**
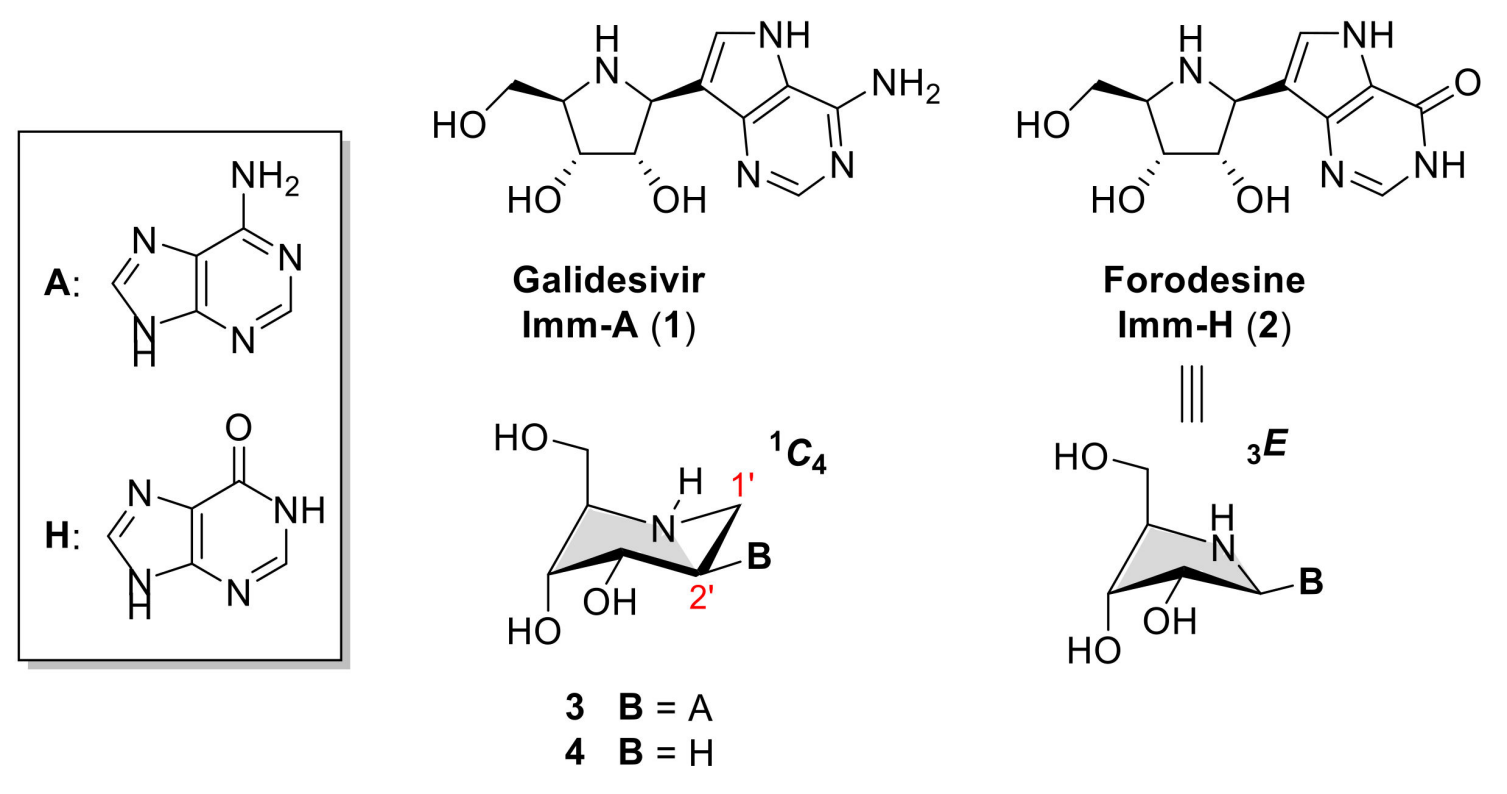
Immucillins A and H (**1** and **2**) and their conformationally restricted mimics **3** and **4**.

The modulation of the activity of carbohydrate processing enzymes represents an important therapeutic target, given the involvement of these proteins in a plethora of metabolic events causing a variety of diverse pathologies [1,2,3]. Over the last decades, intense efforts have been focused on the identification of inhibitors or enhancers of such enzymes [1,4,5,6,7] with promising therapeutic applications for the treatment of viral infections [8,9], cancer [10], diabetes [11], tuberculosis, lysosomal storage diseases [12], and parasitic protozoa [13]. An illustrative example in this area is represented by the class of iminosugars [4,5,7,14], glycomimetics (carbohydrate analogues) having the ring oxygen replaced by an amino group. Iminosugars are able to mimic the transition state of carbohydrate hydrolysis or transfer [15] (involving, in both cases, an upcoming glycosyl oxycarbenium cation), therefore interfering with the activity of carbohydrate-processing enzymes, such as glycosidases and glycosyltransferases [16]. Some among the most important examples of iminosugars include polyhydroxylated piperidines Miglitol, Miglustat, and Migalastat (Figure 1), which are FDA-approved drugs for the treatment of type 2 diabetes [17], Gaucher [18] and Niemann-Pick type C [19], and Fabry disease [20], respectively. Piperidine iminosugars belonging to the unnatural l-series (l-iminosugars) also exhibit pharmacological properties, such as in the case of l-DGJ [21], l-NBDNJ [22] (Figure 1), and its congeners [23,24]. Similarly, iminosugars with a pyrrolidine skeleton (in both enantiomeric series) have been found to hold excellent glycomimetic properties, as in the case of naturally occurring Radicamine A and B [25], 2,5-dideoxy-2,5-imino-d-mannitol (d-DMDP), 1,4-dideoxy-1,4-imino-d-arabinitol (d-DAB-1) (Figure 1), and their structurally related compounds [26,27]. Polyhydroxylated pyrrolidine scaffolds can also be recognized in the structure of Immucillins, which are chemically stable 9-deazapurine *C*-nucleoside analogues endowed with a variety of therapeutic applications, ranging from the treatment of cancer and autoimmune disorders to parasitic and viral infections [28]. Immucillin-A (**1**, Imm-A, also known as BCX4430 or Galidesivir, Figure 2) has demonstrated to be active in in vivo models against a variety of RNA-dependent RNA polymerases (RdRp)-based pathogens, including Ebola, Marburg, Yellow Fever, and Zika viruses [29,30].

The molecule has exerted a broad-spectrum activity in vitro against more than 20 RNA viruses belonging to nine different families, such as flaviviruses, filoviruses and also bunyaviruses, togaviruses, paramyxoviruses, coronaviruses, and arenaviruses [31]. Lately, clinical studies have been undertaken to explore the safety and antiviral properties of Galidesivir in patients with COVID-19 [32,33]. On the other side, Immucillin H (**2**, Imm-H, also known as Forodesine or Mundesine, Figure 2), which is the result of a rational design process and has been at the core of intense kinetic investigations by Schramm et al., is a picomolar inhibitor of bovine and human purine nucleoside phosphorylases [34,35] (PNP, E.C. 2.4.2.1). PNP is a ubiquitous nucleoside processing enzyme essential for DNA and RNA synthesis and it is involved in the reversible phosphorolysis of purine nucleosides to the corresponding bases and (deoxy)ribose-α-1-phosphate. Based on the observation that PNP inhibition stops the growth of activated T-cells, Immucillin-H has been identified as a key candidate for the treatment of leukemia and autoimmune diseases [36].

Over the last decades, further design endeavours have led to the identification of a new generation of Imm-H related PNP inhibitors, endowed with an even higher potency than the parent pyrrolidine nucleoside [37]. All these inhibitors shared an increase of conformational freedom degrees compared with Imm-H [37]. With the aim to provide an alternative strategy to PNP inhibitors, we conceived to replace the pyrrolidine scaffold with a conformationally more restricted biomimetic system. Our approach exploits the well-known finding that the quality of host-guest interactions benefits from the increase in the preorganization of the guest, since no entropy penalty is involved in the binding process. As demonstrative examples, conformationally restricted nucleosides bearing a six-membered sugar unit have already been found to hold an excellent biomimetic potential in various therapeutic contexts [38,39], as a result of the resemblance of natural ribofuranosyl nucleosides in their bioactive conformations [40]. Accordingly, following our longstanding interest in the identification of conformationally restricted molecules with biomimetic properties [39,41,42,43,44,45,46], we undertook the synthesis of novel Immucillin-A and Immucillin-H analogues, i.e., polyhydroxylated piperidine nucleosides **3** and **4** (Figure 2). Our goal was to replace the five membered pyrrolidine scaffold of the drug candidates with a biomimetic six-membered unit. Indeed, among the two chair conformations that **3** and **4** are expected to be able to adopt, the ^1^*C*_4_ form mimics the bioactive _3_*E* conformation of Immucillins when bound to the PNP active site [37]. On this basis, this preliminary study is first aimed to tune up the synthetic path using natural nucleobases as model aglycones, in view of the synthesis of the congeners equipped with the more complex 9-deazapurine nucleobases. Indeed, differently from pyrrolidine nucleosides, move of the nucleobase from C1’ to C2’ in piperidine nucleosides, required for Immucillin biomimicry, allows to install natural nucleobases without affecting the structural integrity of the molecule. In addition, the analysis of the conformational preferences of 3 and 4 is herein performed to ascertain the biomimetic properties of this novel class of nucleoside analogues.

## 2. Results and Discussion

**Scheme 1:**
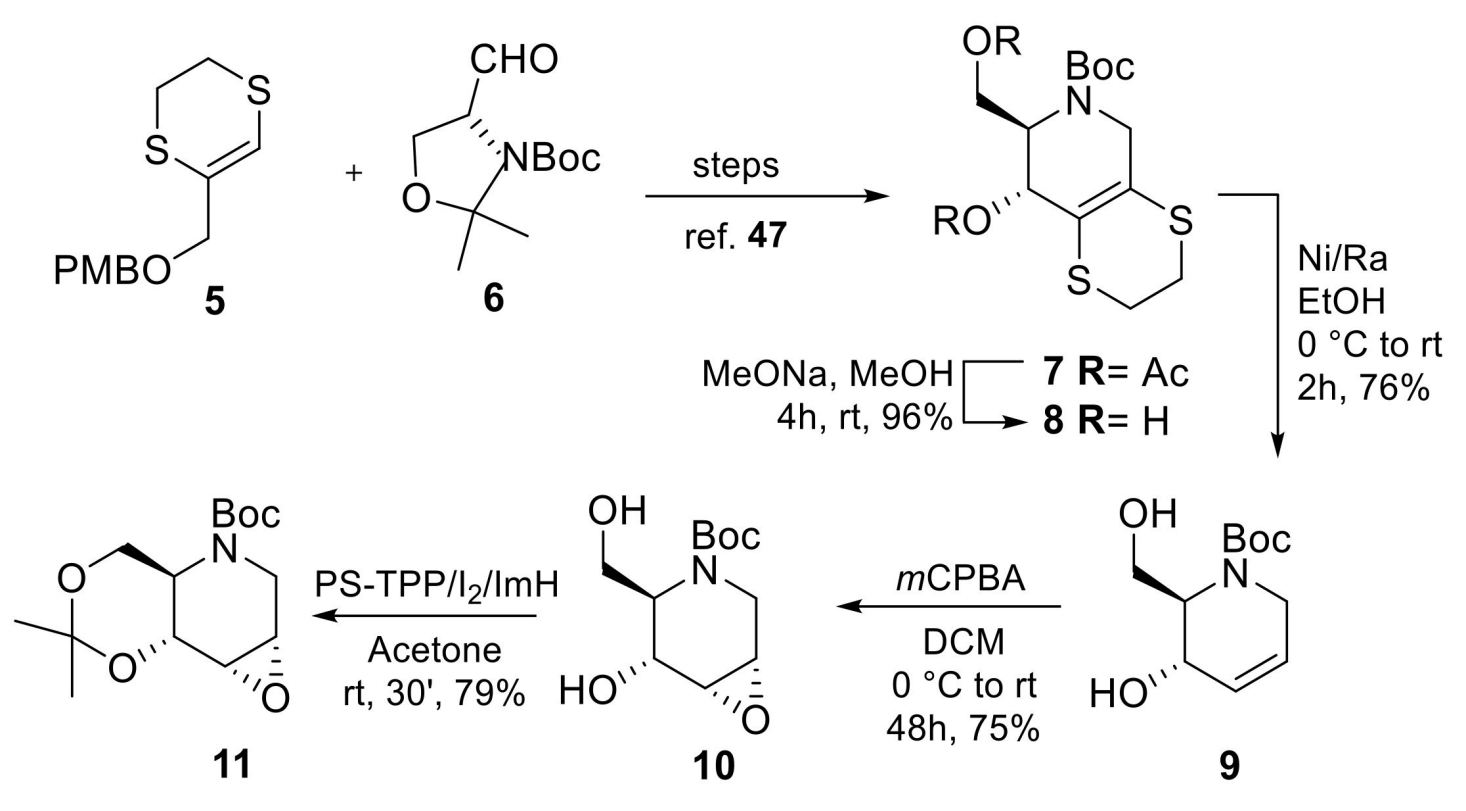
*De novo* approach to cis epoxide **11**.

The synthesis of nucleosides **3** and **4** was achieved from heterocyclic system **7**, in turn obtained in a few steps by dithiin **5** and (*R*)-Garner aldehyde (**6**) (Scheme 1), using a similar synthetic sequence previously enabling the synthesis of unnatural l-iminosugars [22,23,47]. Deacetylation of **7** under Zemplén conditions (NaOMe, MeOH) quantitatively afforded **8** (96%), which was then treated with Raney^®^-Ni in EtOH to cleave the dithioethylene bridge, yielding olefin **9** (76% yield). Stereoselective 4-OH-directed epoxidation of **9**, using *m*CPBA in CH_2_Cl_2_ led, as expected, exclusively to *cis* oxirane **10** (75% yield). In the subsequent ketalization step of OH groups in **10**, we used an alternative procedure to the standard protocol (PTSA, 2,2-dimethoxypropane, 2-methoxypropene) [48], in order to avoid strictly anhydrous conditions, which are typically required to limit by-product formation derived from acid-catalysed oxirane ring opening. Exploiting our longstanding expertise in the field [23,49,50,51,52,53], we chose the procedure involving the use of polymer-supported triphenyl phosphine (PS-TPP)/I_2_/imidazole (ImH) system as the activating agent for the protection reaction, using acetone as the acetonide source. In this case, the reaction involves the activation of acetone by the triphenylphosphonium iodide and the subsequent double attack by the diol to the activated ketone. Challenging for our synthetic target, PS-TPP/I_2_/ImH is also reported to enable epoxide ring opening, to provide the corresponding iodohydrins [54].

However, in our hands, the addition of **10** to a suspension of the premixed activating agent in the anhydrous acetone provided, already after 1 h at room temperature, the corresponding 4’,6’-*O*-isopropylidene derivative **11** with no traces of other byproducts. The pure ketal **11** could be isolated after a simple filtration of the reaction mixture, increasing the synthetic benefits of the procedure.

**Scheme 2:**
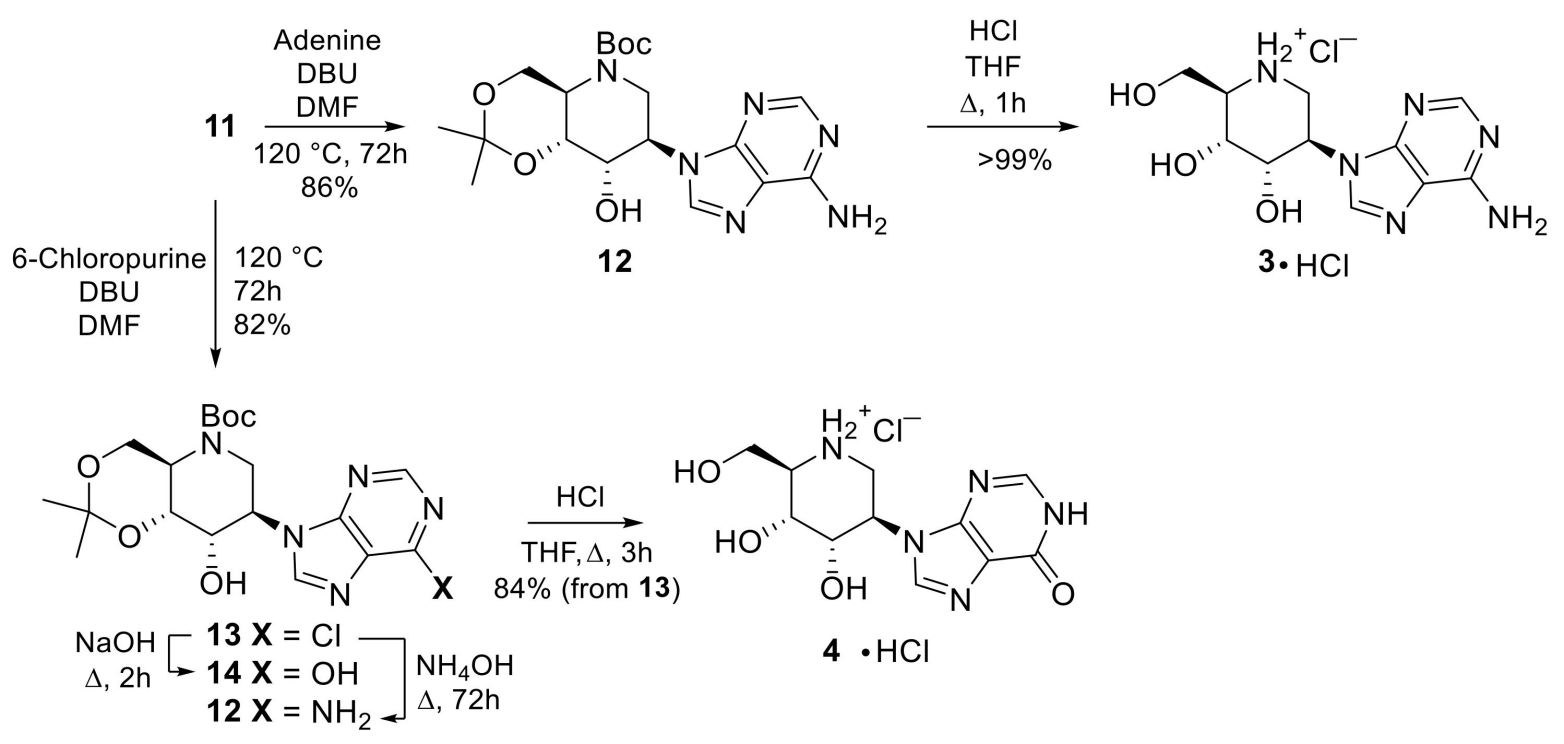
Synthesis of Immucillin H and A analogues **3** and **4**.

With the fully protected epoxide **11** in hand, piperidine nucleosides were eventually obtained, as already reported for similar substrates [42]. By virtue of the locked conformation of compound **11**, the treatment of the latter with DBU and adenine in anhydrous DMF at 120 °C (Scheme 2) only led to the epoxide ring opening by the nucleobase at the desired C2 axial position, affording regioselectively the corresponding protected altritol-like nucleoside **12**, additionally in a very good yield (86%). Under the same coupling conditions, the treatment of **11** with 6-chloropurine provided a mixture of chloropurine and hypoxanthine-containing nucleosides **13** (82%) and **14** (18%), respectively. Alternatively, the same reaction carried out replacing DBU with NaH in DMF at room temperature yielded 6-chloropurine nucleoside as the only observed product. The quantitative conversion of **13** into **14** could be then obtained by the treatment with a refluxing 0.5 M NaOH solution, while the corresponding adenosine derivative **12** could be obtained by the treatment of **13** with conc. NH_4_OH at reflux temperature. Eventually, the subsequent addition of 2 M HCl to crude nucleosides **12** and **14** allowed both isopropylidene and Boc groups removal, providing the corresponding nucleosides **3** and **4** as HCl salts (**3**: > 99% from **12**; **4**: 84% from **13**).

**Figure 3:**
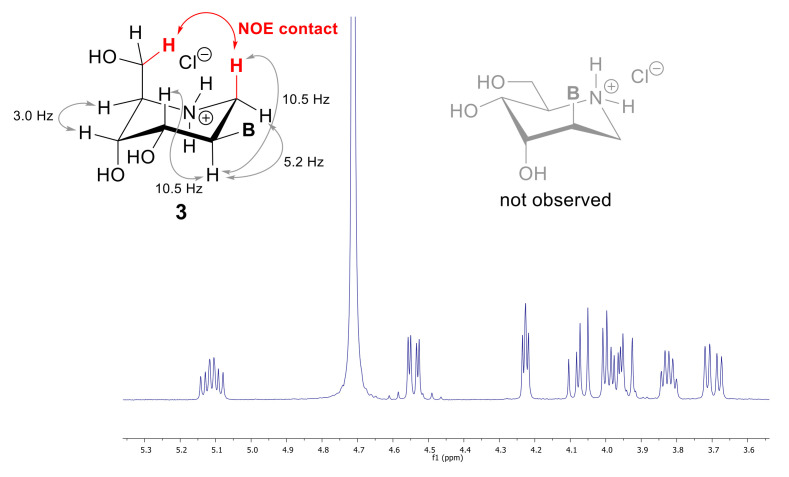
^1^H NMR analysis of **3** HCl.

The NMR analysis of nucleosides **3** and **4** confirmed the formation of N9-C2’ bonds, as revealed by HMBC correlations between H-2’ of the piperidine and the C-4 of purines. Furthermore, 1D and 2D spectra strongly suggested that the desired conformations with equatorially oriented nucleobases were adopted by both nucleosides (Figure 3). Indeed, ^1^H NMR analysis provided large coupling constants between H-1’/H-2’ (10.8 Hz in both cases) and H-2’/H-3’ (**3**: 10.2 Hz; **4**: 10.0 Hz), as well as relatively small coupling constants between H-4’/H-5’ (**3**: 3.1 Hz; **4**: 3.4 Hz), which are responsible for the trans diequatorial interaction between the two protons. On the other hand, the NOESY analysis revealed dipolar interactions between the axially oriented H-1’ and H-6’a (Figure 3).

The observed piperidine conformation is opposite to the ^4^C_1_ form (axially oriented nucleobase) adopted by *altro*-configured nucleosides having a ring oxygen and more generally by the majority of biomimetic hexitol nucleosides [40,55]. Conversely, the conformation of **3** and **4** is in line with those adopted by cyclohexanyl nucleosides, having a methylene group in place of ring heteroatom [42,56]. In agreement with previous studies on both hexitol and cyclohexanyl nucleosides [56], it is conceivable to hypothesize that the conformation of piperidine nucleosides is adopted to relieve the 1,3-diaxial strains involving the nucleobase and hydrogen atoms from C4 and the protonated amino group in the ^4^C_1_ conformer (Figure 3). 

Having equatorially oriented nucleobases, nucleosides **3** and **4** hold the conformational requirements to mimic the _3_*E* pyrrolidine ring puckering of Immucillins H and A. Based on these data, the piperidine core can be therefore considered as an appropriate scaffold for the construction of preorganized Immucillin analogues. 

## 3. Materials and Methods 

### 3.1. Chemistry

All chemicals and solvents were purchased with the highest degree of purity (Sigma-Aldrich, Darmstadt, Germany; Alfa Aesar, Karlsruhe, Germany; VWR, Milan, Italy) and used without further purification. The reactions were monitored by TLC (precoated silica gel plate F254, Merck, Darmstadt, Germany) and the products were detected by exposure to ultraviolet radiation, iodine vapor, and chromic mixture. Column chromatography: Merck Kieselgel 60 (70–230 mesh). The purity of compounds was determined by CHNS analysis and was ≥ 95% in all cases. NMR spectra were acquired on NMR spectrometers operating at 200 MHz (Varian, Palo Alto, California), 400 MHz (Bruker AVANCE, Billerica, Massachusetts, US) or 500 MHz (Varian Inova, Palo Alto, California, US), using CDCl_3_ solutions unless otherwise specified. Coupling constant values (*J*) were reported in Hz, details in Supplementary Materials.

### 3.2. Procedures for the Synthesis of **3**–**13**

Bicyclic Compound **8**. MeONa (16 mg, 0.30 mmol) was added to a stirring solution of **7** [47] (0.12 g, 0.30 mmol) in MeOH (2.0 mL). The mixture was stirred for 4 h at room temperature and then neutralized with a few drops of acetic acid. Then, solvent removal under reduced pressure and chromatography of the crude residue over silica gel (hexane/EtOAc = 6/4) provided pure **2** (92 mg, 96% yield) as a colorless oil. [α]^25^_D_ + 47.2 (*c* 0.22, MeOH). ^1^H NMR (200 MHz): 1.48 (s, 9H), 1.68 (bs 2H), 3.19–3.26 (m, 4H), 3.56–3.68 (m, 3H), 3.90 (bs, 1H), 4.19–4.32 (m, 1H), 4.48–4.56 (m, 1H). ^13^C NMR (50 MHz): 27.8, 28.2, 44.8, 58.3, 60.6, 68.0, 81.0, 119.9, 121.5, 145.9, 155.7 ppm. Anal. calcd for C_13_H_21_NS_2_O_4_: C 48.88, H 6.63, N 4.38, S 20.07. Found: C 48.98, H 6.61, N 4.39, S 20.02.

Diol **9**. To a suspension of Raney-Ni (W2) (0.90 g, wet) in EtOH (1 mL) a solution of bicycle piperidine **8** (90 mg, 0.28 mmol) in the same solvent (3 mL) at 0 °C was added. The suspension was stirred for 2 h at room temperature, then the solid was filtered off and washed with EtOH. The filtrate was concentrated under reduced pressure providing the crude residue whose chromatography over silica gel (hexane/acetone = 6/4) gave pure **9** (49 mg, 76% yield) as a colorless oil. [α]^25^_D_ + 87.8 (*c* 0.85, CHCl_3_). NMR data for **9** were consistent with those reported elsewhere [57]. Anal. calcd for C_11_H_19_NO_4_: C 57.63, H 8.35, N 6.11. Found: C 57.73, H 8.83, N 6.10.

Epoxide **10**. To a stirred solution of diol **9** (49 mg, 0.21 mmol) in anhydrous CH_2_Cl_2_ (2 mL), *m*-CPBA (43 mg, 0.25 mmol) was added at 0 °C. The mixture was stirred for 48 h at room temperature and then aq. NaHCO_3_ was added and the mixture was extracted with CH_2_Cl_2_. The organic layer was dried (Na_2_SO_4_) and the solvent evaporated under reduced pressure. Chromatography of the crude residue over silica gel (hexane/EtOAc = 1:9) afforded pure **10** (40 mg, 75% yield): oily, [α]^25^_D_ + 14.3 (*c* 1.0, CHCl_3_). ^1^H NMR (400 MHz): δ 1.46 (s, 9H), 2.20 (bs, D_2_O exchange, 2H), 3.31 (bs, 1H), 3.39 (bd, *J* = 3.8, 1H), 3.46 (t, *J* = 4.5, 1H), 3.59 (d, *J* = 8.0, 11.3, 1H), 3.68 (dd, *J* = 6.0, 11.3, 1H), 3.94 (dd, *J* = 1.4, 4.5, 1H), 4.18 (bt, *J* = 6.0, 1H), 4.28 (bs, 1H). ^13^C NMR (100 MHz): 24.3, 47.5, 48.3, 52.9, 56.4, 58.5, 7.2, 76.9, 152.4 ppm. Anal. calcd for C_11_H_19_NO_5_: C 53.87, H 7.81, N 5.71. Found: C 53.96, H 7.79, N 5.72.

Protected Epoxide **11**. To a magnetically stirred solution of polymer supported triphenylphosphine (PS-TPP; 100–200 mesh, extent of labeling: ~3 mmol/g triphenylphosphine loading) (80 mg, ~0.24 mmol) in anhydrous acetone (0.5 mL) at room temperature, a solution of I_2_ (60 mg, 0.24 mmol) in the same solvent (0.7 mL) was added dropwise in the dark and under dry N_2_ atmosphere. Subsequently imidazole (32 mg, 0.48 mmol) was added and after 15 min **10** (40 mg, 0.16 mmol) was added in one portion to the suspension. TLC monitoring showed the complete consumption of starting sugar within 10 min. The resulting mixture was filtered and the solvent removed at room temperature under reduced pressure, affording **11** (36 mg, 79% yield) as a colorless oil. [α]^25^_D_ + 1.2 (*c* 2.0, CHCl_3_). ^1^H NMR (500 MHz, acetone-*d_6_*): δ 1.35 (s, 3H), 1.46 (s, 9H), 1.52 (s, 3H), 3.26 (d, *J* = 4.8, 1H), 3.31 (td, *J* = 4.8, 10.7 1H), 3.41–3.44 (m, 1H), 3.76 (d, *J* = 15.3, 1H), 3.83 (dd, *J* = 2.0, 15.3, 1H), 4.15 (t, *J* = 10.7, 1H), 4.23 (dd, *J* = 4.8, 10.7, 1H), 4.37 (d, *J* = 10.7, 1H). ^13^C NMR (125 MHz, acetone-*d_6_*): 18.6, 27.5, 29.7, 42.6, 51.0, 51.8, 62.4, 69.5, 79.8, 98.9, 154.4 ppm. Anal. calcd for C_14_H_23_NO_5_: C 58.93, H 8.13, N 4.91. Found: C 59.06, H 8.1, N 4.92.

Nucleoside **12**. Adenine (36 mg, 0.26 mmol) and epoxide **11** (35 mg, 0.12 mmol) were suspended in anhydrous DMF (0.9 mL) for 15 min, at room temperature under Ar atmosphere. Then, 1,8-diazabicyclo[5.4.0]undec-7-ene (DBU, 0.26 mmol, 39 uL) was added and the resulting mixture was heated at 90 °C and stirred for 72 h. The reaction mixture was cooled to room temperature, quenched with sat.aq. NH_4_Cl, and concentrated under reduced pressure. The crude residue was extracted with EtOAc and washed with brine. The organic layers were dried (Na_2_SO_4_) and the solvent evaporated under reduced pressure. Chromatography of the crude residue over silica gel (EtOAc:MeOH = 9:1) gave pure **12** (43 mg, 86% yield): oily, [α]^25^_D_ − 21.2 (*c* 1.0, MeOH). ^1^H NMR (400 MHz, CD_3_OD): δ 1.44 (s, 3H), 1.47 (s, 9H), 1.60 (s, 3H), 3.78 (td, *J* = 4.7, 10.5, 1H), 3.98 (dd, *J* = 5.2, 13.9, 1H), 4.03–4.12 (m, 2H), 4.34 (bt, *J* = 2.7, 1H), 4.38 (dd, *J* = 4.7, 10.5 Hz, 1H), 4.60–4.65 (m, 2H), 8.21 (s, 1H), 8.22 (s, 1H).^13^C NMR (100 MHz, CD_3_OD): 19.6, 28.5, 29.4, 42.9, 59.8, 63.9, 70.2, 70.9, 82.4, 100.7, 120.2, 141.5, 150.8, 153.8, 156.6, 157.5 ppm. Anal. calcd for C_19_H_28_N_6_O_5_: C 54.27, H 6.71, N 19.99. Found: C 54.27, H 6.73, N 20.05.

Nucleoside **13.** 6-Chloropurine (31 mg, 0.20 mmol) and epoxide **11** (25 mg, 0.09 mmol) were suspended in anhydrous DMF (0.5 mL) at room temperature under Ar atmosphere. After 15 min, 1,8-diazobicyclo[5.4.0]undec-7-ene (DBU, 30 μL, 0.20 mmol) was added and the reaction mixture was heated at 120 °C and stirred for 72 h. Then, the reaction mixture was cooled to room temperature, quenched with NH_4_Cl, and concentrated under reduced pressure. The crude residue was extracted with DCM and washed with brine. The organic layers were dried (Na_2_SO_4_) and the solvent evaporated under reduced pressure. Flash chromatography of the crude residue over silica gel (AcOEt) gave pure **13** (32 mg, 82% yield): oily, [α]^25^_D_ + 3.3 (*c* 0.33, acetone). ^1^H NMR (CDCl_3_, 500 MHz): δ 1.42 (s, 3H), 1.45 (s, 9H), 1.50 (s, 3H), 3.54 (bs, 1H), 3.67 (td, *J* = 10.5, 4.9 Hz, 1H), 3.89 (dd, *J* = 14.3, 4.4 Hz, 1H), 4.12 (t, *J* = 11.0 Hz, 1H), 4.17 (dd, *J* = 3.0, 10.5 Hz, 1H), 4.27 (dd, *J* = 14.3, 6.0 Hz, 1H), 4.36 (t, *J* = 2.9 Hz, 1H), 4.51 (dd, *J* = 11.5, 4.9 Hz, 1H), 4.62–4.64 (m, 1H), 7.87 (s, 1H), 8.32 (s, 1H). ^13^C NMR (100 MHz): 19.6, 28.5, 29.3, 42.8, 49.6, 55.9, 63.0, 68.6, 69.7, 81.7, 99.4, 120.3, 137.0, 150.6, 152.6, 154.7 ppm. Anal. calcd for C_19_H_26_ClN_5_O_5_: C 51.88, H 5.96, Cl 8.06, N 15.92. Found: C 51.77, H 5.94, Cl 8.09, N 15.97.

Adenosine analogue **3.** Procedure A (from **12**). 2 M HCl (11.6 mL) was added to a solution of **12** (40 mg, 0.09 mmol) in THF (0.6 mL) and the reaction mixture was heated to reflux temperature for 1 h. Removal of the volatiles under reduced pressure and subsequent trituration with Et_2_O afforded **3** as hydrochloride salt (30 mg, quant.). Procedure B (from **13**). Compound **13** (15 mg, 0.03 mmol) was treated with 13 m NH_4_OH (4.5 mL). The mixture was added to a steal bomb reactor heated to reflux temperature for 72 h. The reaction was quenched by the addition of a few drops of HCl (1n) and concentrated under reduced pressure. The crude residue was then diluted with CHCl_3_ : MeOH = 8:2 and filtered under a silica pad. Volatiles were removed under reduced pressure to obtain **12** a white solid. As described in procedure A, THF (2 mL) and HCl 2 m (0.6 mL) were then added and the solution was heated to reflux temperature for 1 h. Removal of the solvents under reduced pressure and the subsequent trituration of the solid with Et_2_O gave pure **3** as hydrochloride salt (7.0 mg, 65% yield over two steps). Data for **3**: white solid, [α]^25^_D_ −8.25 (*c* 0.14, H_2_O). ^1^H NMR (400 MHz, D_2_O): δ 3.76 (dd, *J* = 5.2, 13.4, 1H), 3.90 (dt, *J* = 3.5, 8.8, 1H), 4.03 (dd, *J* = 10.5, 13.4, 1H), 4.07 (dd, *J* = 3.5, 12.8, 1H), 4.16 (dd, *J* = 8.8, 12.8, 1H), 4.31 (t, *J* = 3.5 Hz, 1H), 4.62 (dd, *J* = 3.5, 10.5, 1H), 5.19 (td, *J* = 5.2, 10.5, 1H), 8.38 (s, 1H), 8.39 (s, 1H). ^13^C NMR (100 MHz, D_2_O): 39.7, 53.3, 55.8, 59.9, 65.9, 66.9, 118.9, 144.2, 144.4, 148.6, 149.9 ppm. Anal. calcd for C_11_H_17_ClN_6_O_3_: C 41.71, H 5.41, Cl 11.19, N 26.53. Found: C 41.56, H 5.39, Cl 11.24, N 26.63.

Hypoxanthine analogue **4.** Nucleoside **13** (15 mg, 0.03 mmol) was refluxed for 2 h in a 0.5 n aq NaOH (0.5 mL). Then, the reaction mixture was cooled to 0 °C and 0.5 n HCl was carefully added (0.8 mL). The solution was evaporated under reduced pressure. The crude was then dissolved in THF (0.2 mL) and then 2 m HCl (0.5 mL) was added. The reaction mixture was heated to reflux temperature for 3 h. Then, the solvent was evaporated under reduced pressure and the subsequent trituration of the solid with Et_2_O gave pure **4** as hydrochloride salt (8.0 mg, 84%). [α]^25^_D_ + 2.8 (*c* 0.14, H_2_O). ^1^H NMR (400 MHz, D_2_O): δ 3.74 (dd, *J* = 5.1, 13.3, 1H), 3.89 (m, 1H), 4.02 (dd, *J* = 10.8, 13.3, 1H), 4.05 (dd, *J* = 3.4, 12.8, 1H), 4.15 (dd, *J* = 8.9, 12.8, 1H), 4.30 (t, *J* = 3.4 Hz, 1H), 4.61 (dd, *J* = 3.3, 10.0, 1H), 5.18 (td, *J* = 5.1, 10.0, 1H), 8.40 (*s*, 1H), 8.42 (*s*, 1H). ^13^C NMR (100 MHz, D_2_O): 35.8, 49.1, 51.8, 55.9, 62.0, 62.8, 115.6, 123.4, 138.6, 140.5, 144.3 ppm. Anal. calcd for C_11_H_16_ClN_5_O_4_: C 41.58, H 5.08, Cl 11.16, N 22.04. Found: C 41.49, H 5.10, Cl 11.18, N 22.04.

## 4. Conclusions

The synthesis of piperidine nucleosides **3** and **4**, acting as conformationally restricted analogues of Immucillins H and A, has been herein reported. Our approach exploits our consolidated strategy, involving the synthetic manipulation of coupling product **7** starting from our three-carbon homologating agent **5**. The stereoselective preparation of *cis* epoxide **11** and the subsequent coupling reaction in two alternative approaches with model nucleobases have enabled access to piperidine nucleosides **3** and **4** with the desired d-*altro* configuration. NMR analysis demonstrated that both compounds resemble the bioactive conformation of Immucillins H and A, as they adopt iminosugar conformations with equatorially oriented nucleobases. Further studies will be devoted to explore the suitability of our approach to introduce a variety of other heterocyclic bases for SAR analysis. Particularly, synthetic studies aimed to incorporate Immucillin-mimicking 9-deazapurine nucleobases and in vitro assays, aimed to ascertain the pharmacological potential of all the synthesised nucleosides, are currently ongoing and will be published elsewhere.

## Data Availability

The data presented in this study are available within the article and in the Supplementary Materials section.

## Supplementary Materials

Figure S1: 1H and 13C NMR spectra of compound 8; Figure S2: 1H and 13C NMR spectra of compound 10; Figure S3: 1H and 13C spectra of compound 11; Figure S4: 1H and 13C NMR spectra of compound 12; Figure S5: 1H and 13C NMR spectra of compound 13; Figure S6: 1H and 13C NMR spectra of compound 3; Figure S7: 1H and ^13^C NMR spectra of compound **4.**
