# Supplementary material for: Synthesis of Piperidine Nucleosides as Conformationally Restricted Immucillin Mimics
Source: Molecules. 2021 Mar 16;26(6):1652. doi: 10.3390/molecules26061652 (PMC8001838; doi:10.3390/molecules26061652)
Supplement: Supplementary file 1 [file molecules-26-01652-s001.zip › molecules-1142603-supplementary.pdf]

SUPPORTING INFORMATION

Synthesis of Piperidine Nucleosides as Conformationally Restricted Immucillin Mimics

Maria De Fenza [1], Anna Esposito [1], Daniele D'Alonzo [1] and Annalisa Guaragna [2,*]

[1] Department of Chemical Sciences, University of Naples Federico II, Via Cintia, 80126 Naples, Italy

[2] Department of Chemical, Materials and Production Engineering, University of Naples Federico II, Piazzale V. Tecchio 80, 80125 Naples, Italy

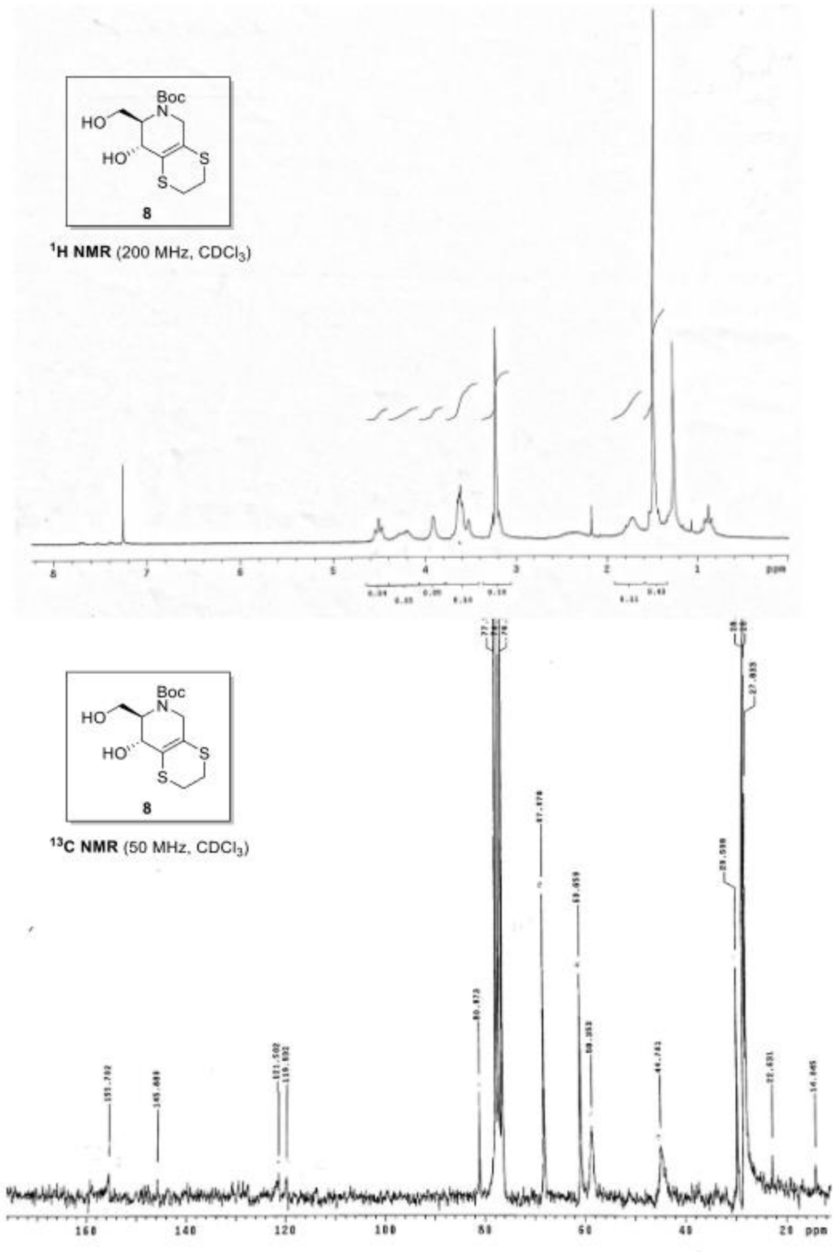

Figure S1. ^{1}H and ^{13}C NMR spectra of compound **8**.

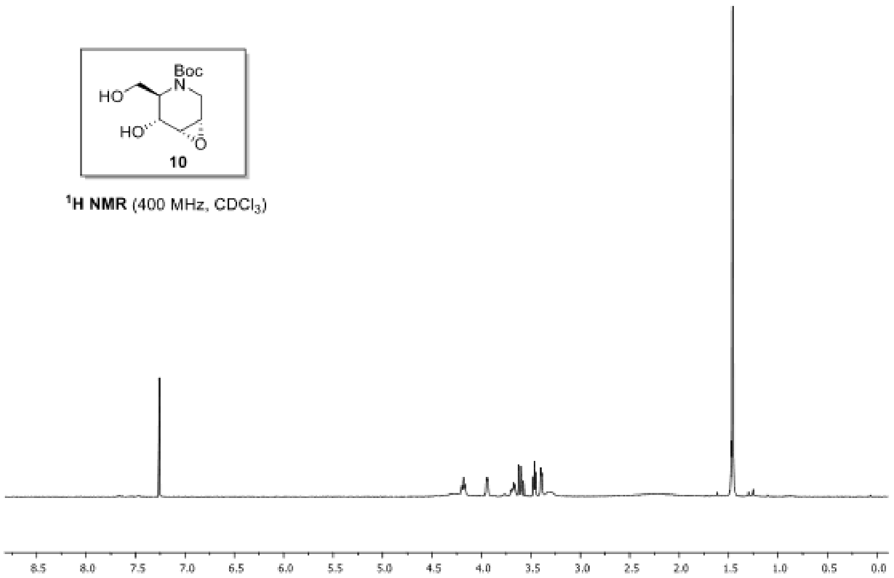

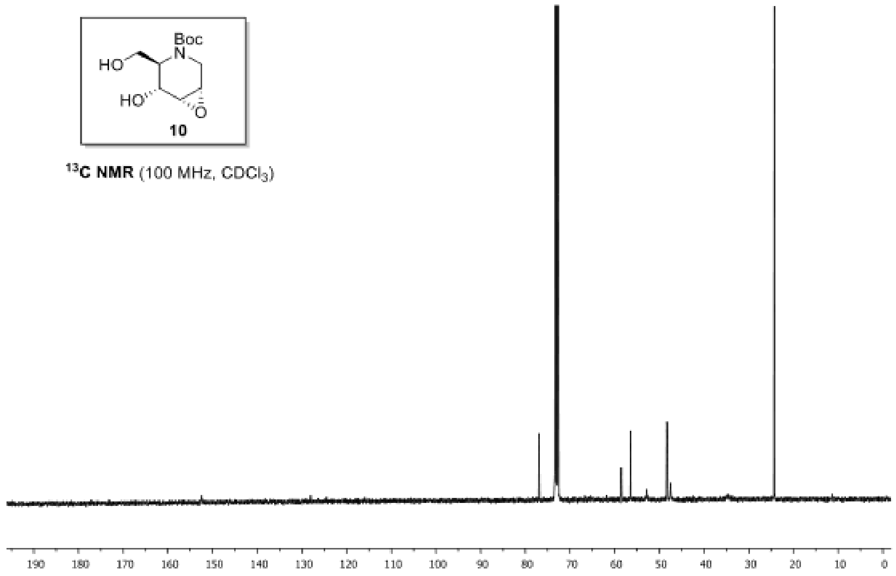

Figure S2. ^{1}H and ^{13}C NMR spectra of compound **10.**

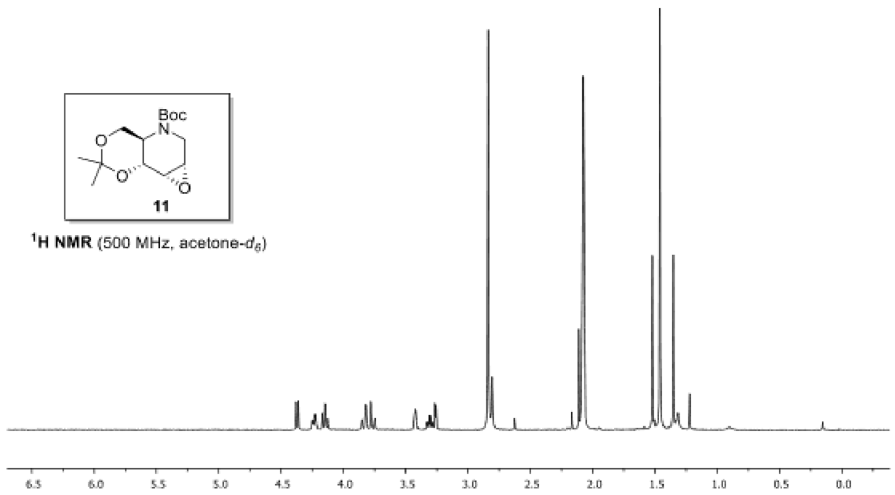

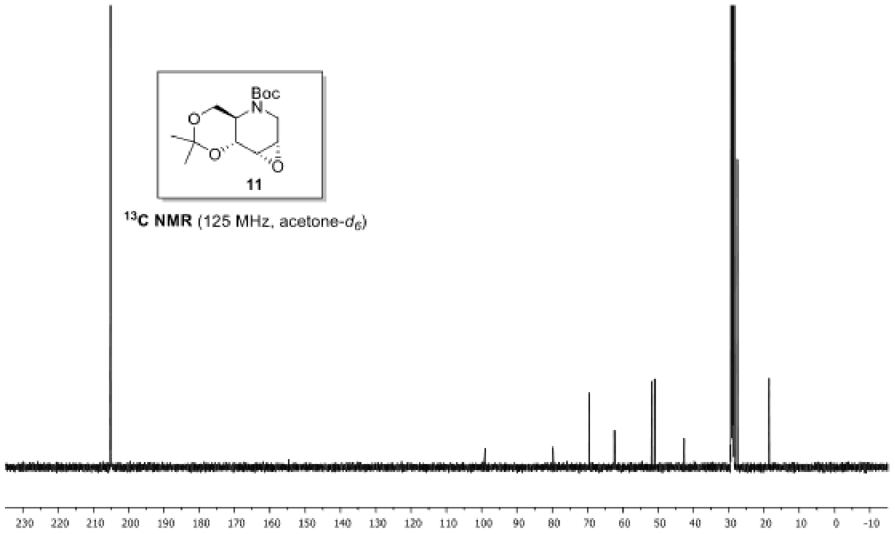

Figure S3. ^{1}H and ^{13}C NMR spectra of compound **11.**

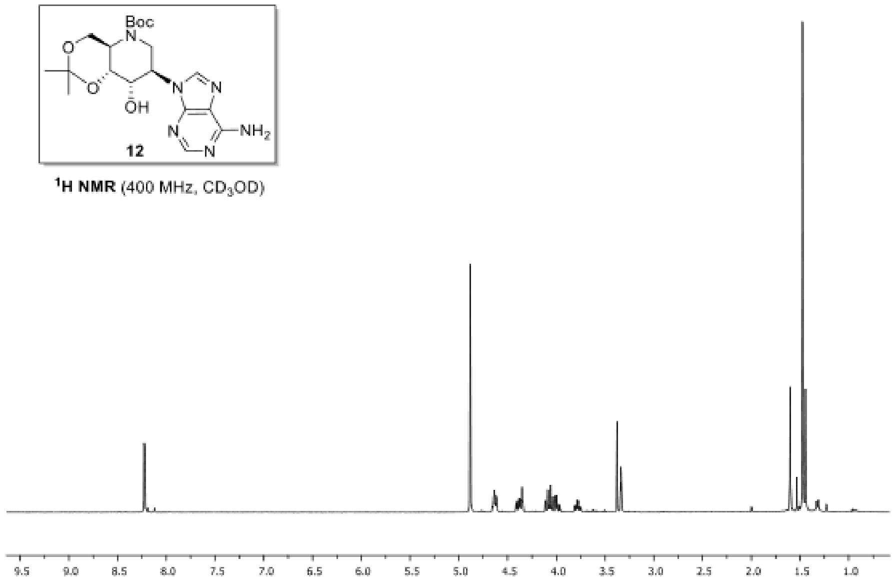

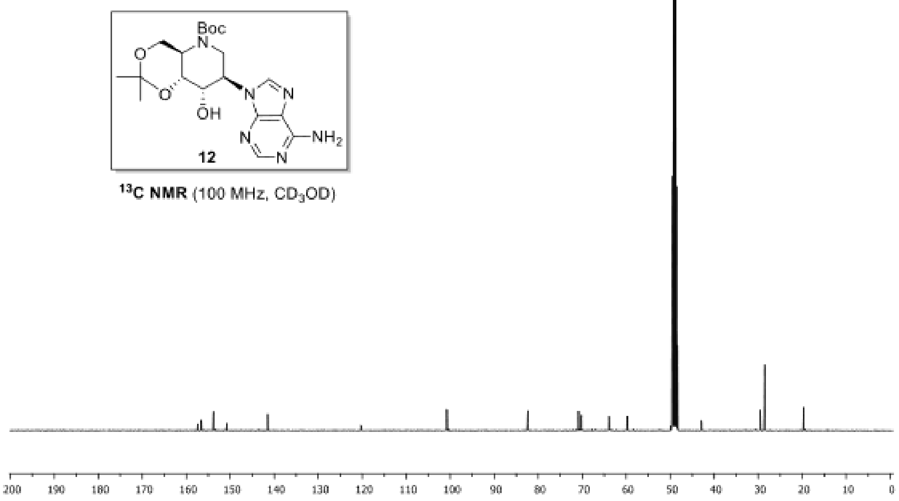

Figure S4. ^{1}H and ^{13}C NMR spectra of compound **12.**

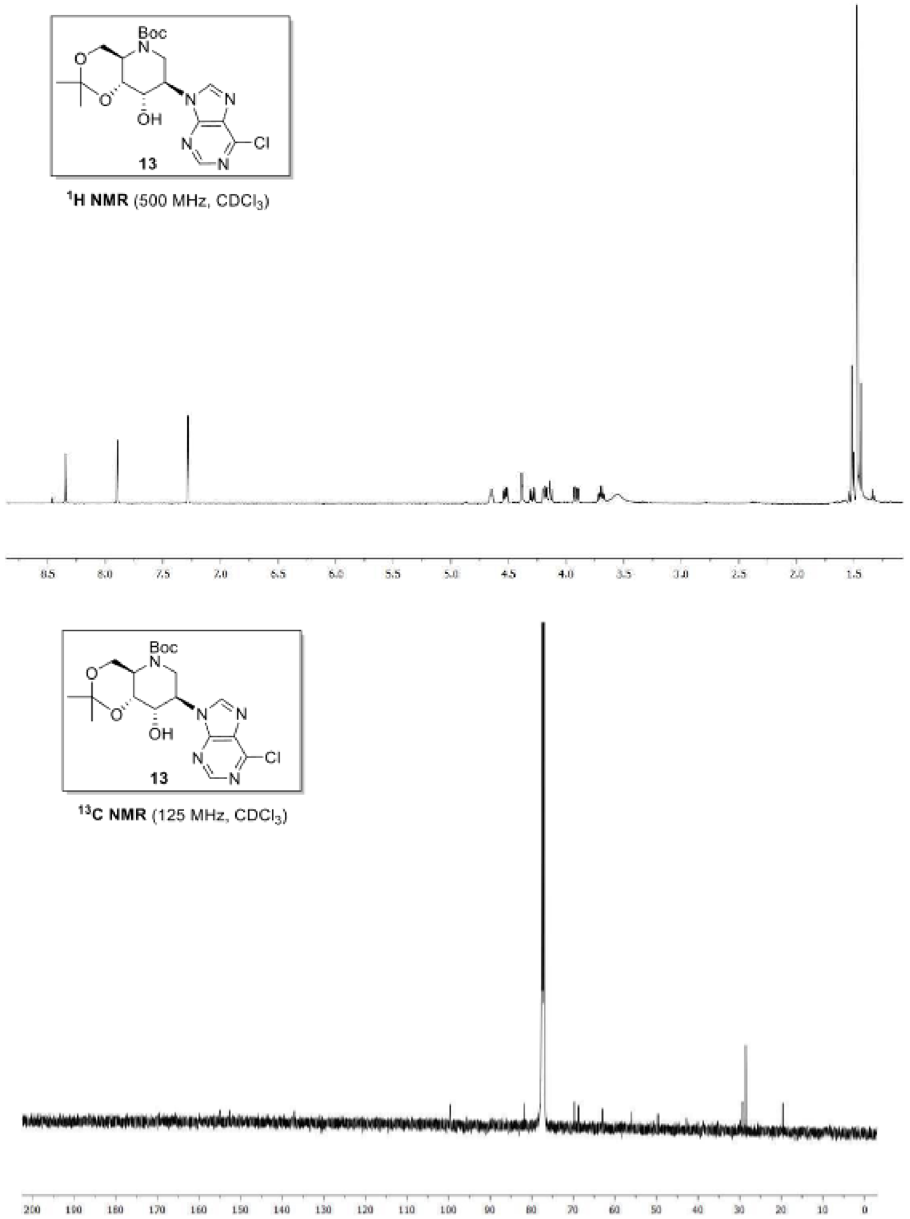

Figure S5. ^{1}H and ^{13}C NMR spectra of compound **13.**

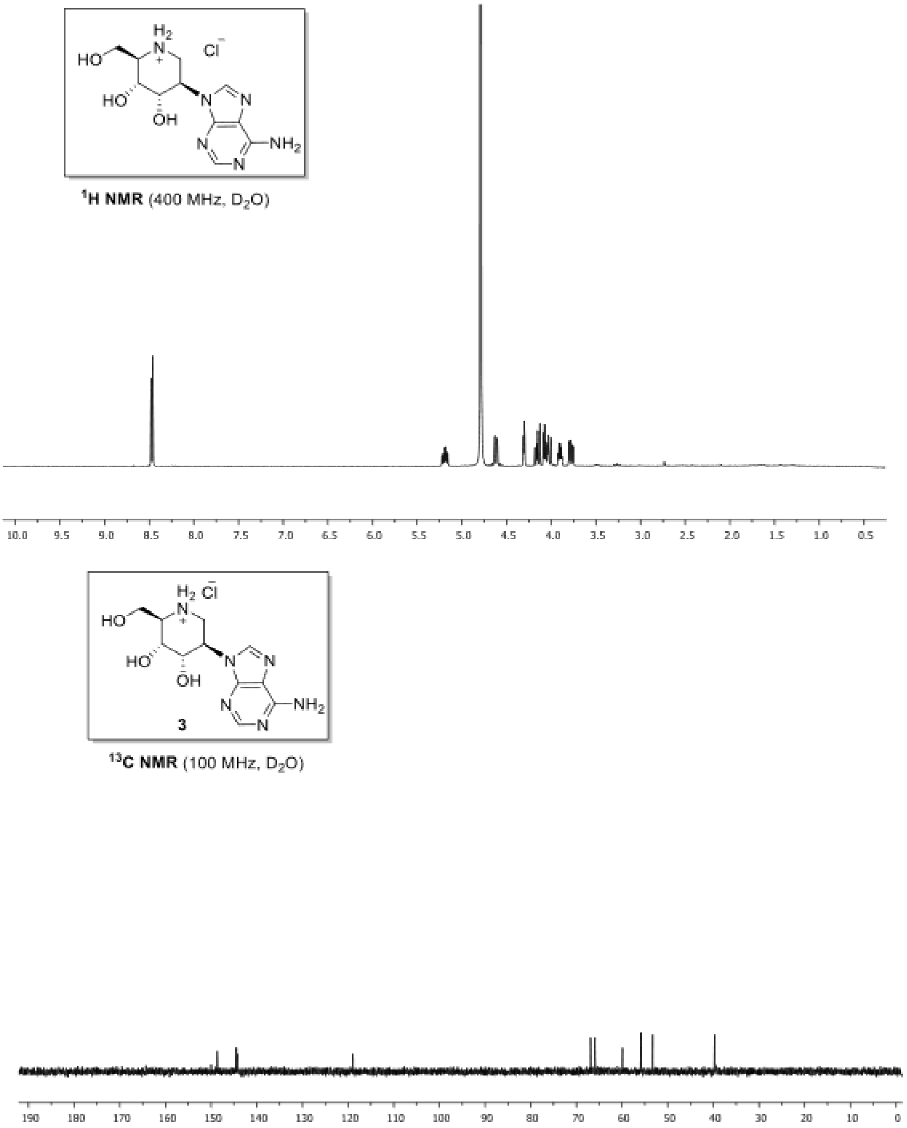

Figure S6. ^{1}H and ^{13}C NMR spectra of compound **3.**

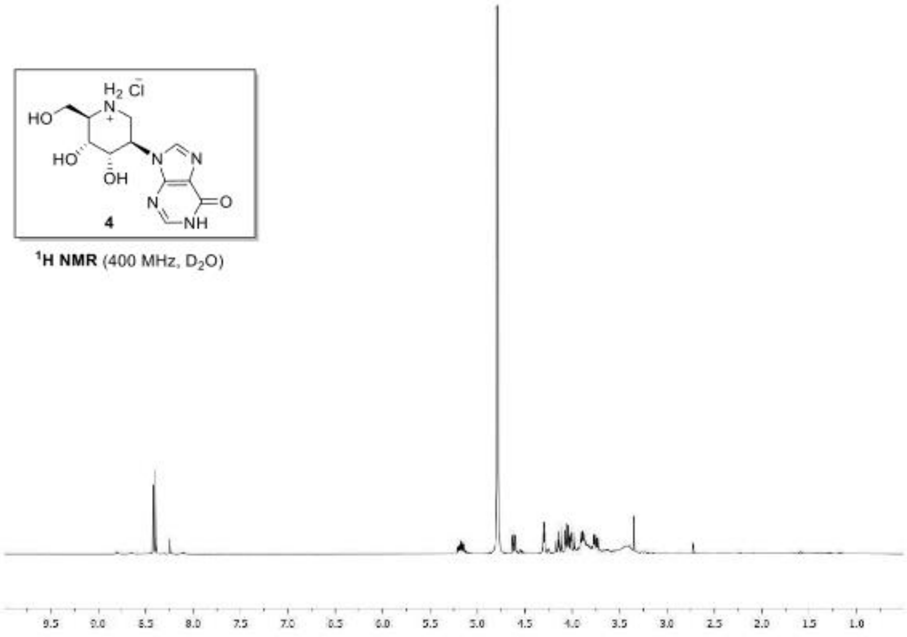

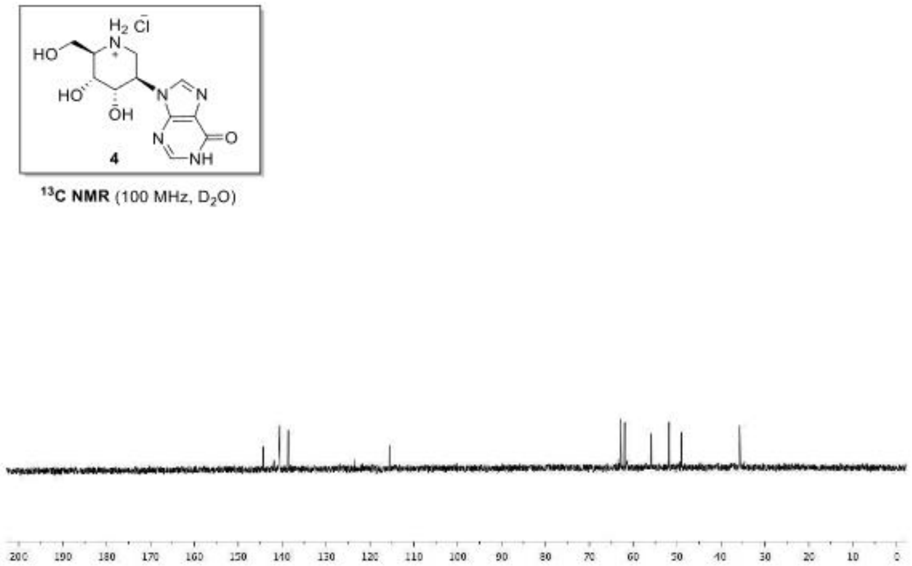

Figure S7. ^{1}H and ^{13}C NMR spectra of compound **4.**
